# The Use of Pentaploid Crosses for the Introgression of *Amblyopyrum muticum* and D-Genome Chromosome Segments Into Durum Wheat

**DOI:** 10.3389/fpls.2019.01110

**Published:** 2019-09-18

**Authors:** Manel Othmeni, Surbhi Grewal, Stella Hubbart-Edwards, Caiyun Yang, Duncan Scholefield, Stephen Ashling, Amor Yahyaoui, Perry Gustafson, Pawan K. Singh, Ian P. King, Julie King

**Affiliations:** ^1^Nottingham BBSRC Wheat Research Centre, Division of Plant and Cop Sciences, School of Biosciences, The University of Nottingham, Sutton Bonington Campus, Loughborough, Leicestershire, United Kingdom; ^2^International Maize and Wheat Improvement Center (CIMMYT) Mexico, Mexico City, Mexico; ^3^Division of Plant Sciences, University of Missouri, Columbia, MO, United States

**Keywords:** durum wheat, pentaploid crosses, *Amblyopyrum muticum*, introgression, *in situ* hybridization, Kompetitive Allele-Specific PCR markers

## Abstract

The wild relatives of wheat provide an important source of genetic variation for wheat improvement. Much of the work in the past aimed at transferring genetic variation from wild relatives into wheat has relied on the exploitation of the *ph1b* mutant, located on the long arm of chromosome 5B. This mutation allows homologous recombination to occur between chromosomes from related but different genomes, e.g. between the chromosomes of wheat and related chromosomes from a wild relative resulting in the generation of interspecific recombinant chromosomes. However, the *ph1b* mutant also enables recombination to occur between the homologous genomes of wheat, e.g. A/B, A/D, B/D, resulting in the generation of wheat intergenomic recombinant chromosomes. In this work we report on the presence of wheat intergenomic recombinants in the genomic background of hexaploid wheat/*Amblyopyrum muticum* introgression lines. The transfer of genomic rearrangements involving the D-genome through pentaploid crosses provides a strategy by which the D-genome of wheat can be introgressed into durum wheat. Hence, a pentaploid crossing strategy was used to transfer D-genome segments, introgressed with either the A- and/or the B-genome, into the tetraploid background of two durum wheat genotypes Karim and Om Rabi 5 in either the presence or absence of different *Am. muticum* (2n = 2x = 14, TT) introgressions. Introgressions were monitored in backcross generations to the durum wheat parents *via* multi-color genomic *in situ* hybridization (mc-GISH). Tetraploid lines carrying homozygous D-genome introgressions, as well as simultaneous homozygous D- and T-genome introgressions, were developed. Introgression lines were characterized *via* Kompetitive Allele-Specific PCR (KASP) markers and multi-color fluorescence *in situ* hybridization (FISH). Results showed that new wheat sub-genomic translocations were generated at each generation in progeny that carried any *Am. muticum* chromosome introgression irrespective of the linkage group that the segment was derived from. The highest frequencies of homologous recombination were observed between the A- and the D-genomes. Results indicated that the genotype Karim had a higher tolerance to genomic rearrangements and T-genome introgressions compared to Om Rabi 5. This indicates the importance of the selection of the parental genotype when attempting to transfer/develop introgressions into durum wheat from pentaploid crosses.

## Introduction

The most important cultivated *Triticum* species are hexaploid bread wheat (2n = 2x = 42; AABBDD, *Triticum aestivum* L. ssp. *aestivum*) and tetraploid durum wheat (2n = 2x = 28; AABB, *Triticum turgidum* L. ssp. *durum*). Tetraploid wheat arose 500,000 years ago from a cross between the wild ancestors of the A-genome, *Triticum urartu* Thum ex. Gandil (2n = 2x = 14; A^u^A^u^) (Feldman and Levy, 2005), and the B-genome from an *Aegilops speltoides*-like progenitor (Haider, 2013). After domestication, a spontaneous cross of tetraploid wheat as the female parent with the goat grass *Ae. tauschii* Coss. (2n = 2x = 14; DD) approximately 8,000 years ago gave rise to hexaploid bread wheat (Kihara, 1944; McFadden and Sears, 1944, Matsuoka and Nasuda, 2004). The addition of the D-genome to hexaploid wheat conferred baking characteristics and a wide climatic adaptation compared to durum wheat (Zohary et al., 1969) resulting in bread wheat becoming one of the most widely grown crops due to its high yields and nutritional and processing qualities (Shewry and Hey, 2015).

Despite the relatively small growing area (8%) and lower annual production compared to bread wheat, durum wheat remains a major crop in the Mediterranean basin where about 75% of the world’s durum wheat is produced (Li et al., 2013; Kabbaj et al., 2017) although Europe and North Africa are also the largest importers of durum wheat (Bonjean et al., 2016). According to data from the International Grain Council, durum wheat production has shown annual fluctuations, largely attributable to abiotic and biotic stresses, e.g., in the Mediterranean area, crops are often exposed to environmental stresses such as high temperature and drought during grain filling (Nazco et al., 2012). Breeding programs have greatly improved durum wheat yield and quality (Magallanes-López et al., 2017). However, the incorporation of new alleles into wheat germplasm is considered essential for the continued improvement of durum wheat productivity.

Wheat is related to a large number of other species, many of which are wild and uncultivated. These wild relatives provide a vast and largely untapped reservoir of genetic variation for agronomically important traits (Friebe et al., 1996; Jauhar and Chibbar, 1999; Qi et al., 2007; Schneider et al., 2008). The incorporation of these traits into wheat has the potential to increase the yield potential. For example, *Ae. speltoides* has been shown to be insect and disease resistant (Elek et al., 2014) and *Thinopyrum bessarabicum* salt tolerant (King et al., 1997).

Among the wild relatives of wheat, *Am. muticum* (2n = 2x = 14; TT) is an annual, native species of Turkey and Armenia (Kilian et al., 2013). This species has been reported to be resistant to environmental stresses (Iefimenko et al., 2015), powdery mildew (Eser, 1998), and leaf rust (Dundas et al., 2015). The introgression of *Am. muticum* into bread wheat is an ongoing project at the Wheat Research Centre (WRC) at the University of Nottingham (King et al., 2013; King et al., 2017) where 218 genome- wide bread wheat/*Am. muticum* introgressions have been developed covering the seven linkage groups of *Am. muticum* (King et al., 2017). Genomic *in situ* hybridization (GISH) analysis revealed that some of the introgression lines also contained intergenomic rearrangements between the A, B, and D sub-genomes of wheat. These intergenomic recombinants, and particularly those that involve the D-genome, can be transferred into durum wheat. Hybridization between bread and durum wheat leads to the production of a pentaploid hybrid (AABBD) with a chromosomal constitution of 2n = 5x = 35 (Kihara, 1924). Depending on the direction of the backcrosses, pentaploid hybrids have the potential to improve both bread wheat and durum (Eberhard et al., 2010; Martin et al., 2013; Kalous et al., 2015).

This paper describes the introgression of both wheat inter-genome rearrangements involving the D-genome and T-genome segments of *Am. muticum* present in hexaploid wheat/*Am. muticum* introgression (WMI) lines into two durum wheat genotypes using pentaploid crosses. The effect of the presence of the T-genome in the WMI lines, the efficiency of the crossing strategy as well as the choice of the durum wheat are discussed.

## Materials and Methods

### Plant Material

**Figure 1:**
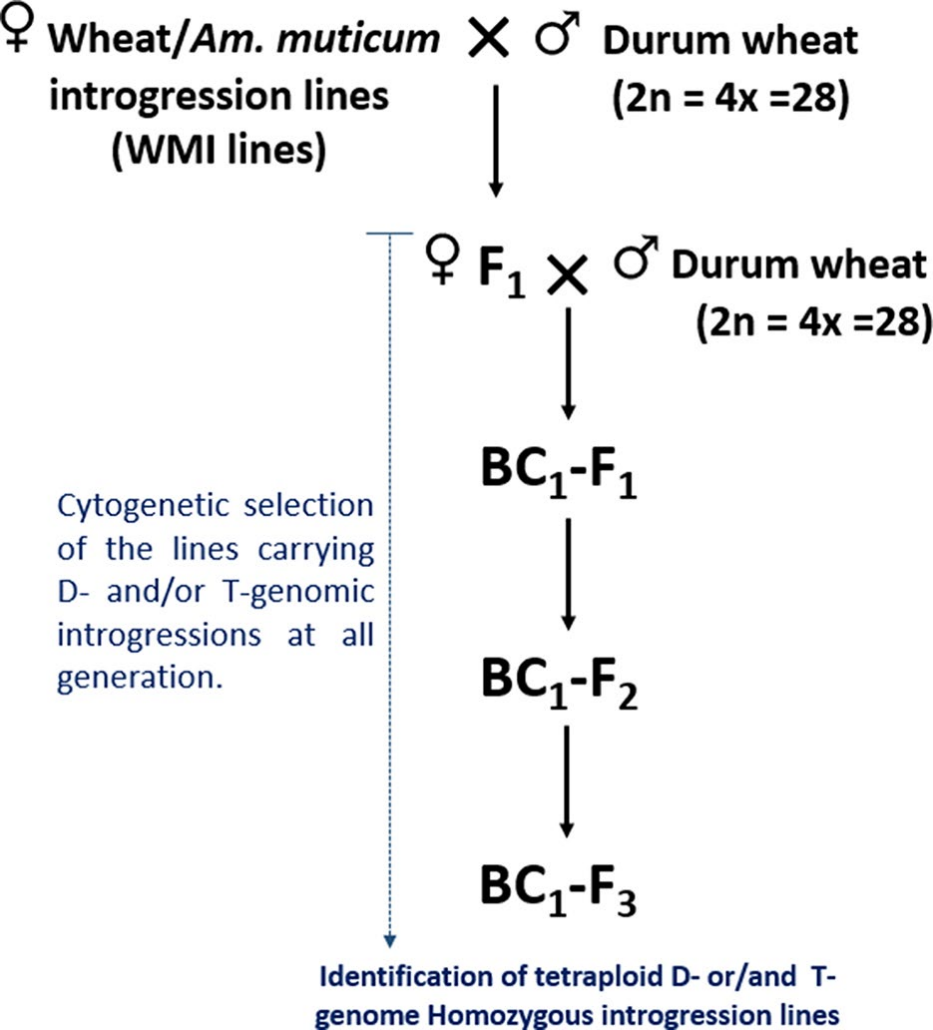
Crossing diagram for the introgression of the D- and T-genome segments identified in WMI lines into durum wheat.

The self-fertilized or back-crossed seed of eight hexaploid wheat/*Am. muticum* introgression lines, designated as WMI (wheat/*Am. muticum* introgression) lines, were obtained from the Nottingham/BBSRC Wheat Research Centre (WRC) (King et al., 2017). The WMI lines were characterized by multi-color genomic *in situ* hybridization (mc-GISH) in the BC_3_ generation and shown to carry wheat inter-genomic rearrangements involving the D-genome. The genome rearrangements were designated by the letter of the genome involved (A, B, or D). An upper case letter designated the larger segment, a lower case letter the smaller segment. In the case of non centromeric translocations, the two letters were separated by a dash (e.g. A-d), whereas for centromeric translocations, a dot was used (e.g., A.D). Four of the WMI lines also carried one to three different large T-genome segments characterized using the Axiom^®^ Wheat-Relative Genotyping Array (King et al., 2017) (**Table1**). Hence, the WMI lines were categorized into two groups, the G-1 lines without a T introgression/chromosome and the G-2 lines carrying a T introgression/chromosome. Four seeds of each line were germinated and screened for the presence of the D-genome introgression using mc-GISH. Lines that retained the introgressions were then used as the female parent in a pentaploid crossing strategy involving two durum wheat genotypes Karim and Om Rabi 5 (**Figure 1**).

**Table 1:** Type and number of the D- genome and T-genome introgressions present in the parental introgression lines and the reference of the WMI lines used in the crosses.

Group	Parental lines	Genome translocation*^No.^	Number of T-genome introgressions	T-genome introgression linkage group	WMI lines used to cross
G-1	BC_3_-F_1_-157-C	A-d*^1^	0	–	BC_4_-F_1_-129
	BC_3_-F_1_-157-D	D-a*^1^	0	–	BC_4_-F_1_-130
	BC_3_-F_1_-157-E	A-d*^1^	0	–	BC_3_-F_2_-130
	BC_3_-F_1_-172-C	D-a*^1^	0	–	BC_3_-F_2_-132
G-2	BC_3_-F_1_-172-E	D-a-b*^1^ + A-d*^1^	3	1T, 3TL, 5T	BC_3_-F_2_-133
	BC_3_-F_1_-177-E	D.a-b*^1^ + A.D*^1^	2	2T, 4T	BC_3_-F_2_-134
	BC_3_-F_1_-244-A	d-A-d*^1^ + D-a*^1^	1	6TS.7TL	BC_3_-F_2_-135
	BC_3_-F_1_-244-B	A-d*^1^	2	1TS.3TL, 6TS.7TL	BC_3_-F_2_-136

NB: *^No.^indicates the number of copies, G1, WMI parental lines without a T-genome segment; G2, WMI parental lines carrying T-genome segments.

### Genomic *In Situ* Hybridization (GISH)

Slides of chromosome spreads were obtained as described in Kato et al. (2004) and King et al. (2017). Mc-GISH of the slides was conducted using the labeled total genomic DNA of the three putative progenitor species of wheat; *T. urartu* (A-genome), *Ae. speltoides* (B-genome) and *Ae. tauschii* (D-genome), as well as *Am. muticum* (T-genome). DNA was extracted from the young leaves using a CTAB method (Zhang et al., 2013) and labeled using the nick translation procedure (Luchniak et al., 2002). Slides were probed with *T. urartu* labeled with Chroma Tide Alexa Fluor 488-5-dUTP (Invitrogen; C11397; green), *Ae. tauschii* with Alexa Fluor 594-5-dUTP (Invitrogen; C11400; red), *Am. muticum* with Alexa Fluor 546 (Invitrogen; C11401; yellow) and the genomic DNA of *Ae. speltoides* fragmented to 300–500bp (using a heat block for 15 min at 110°C) used as a blocking DNA in a ratio of 1:1:2:30.

For the detection of T-genome introgression alone some of the lines were probed by single color GISH using the labeled *Am. muticum* genomic DNA with Chroma Tide Alexa Fluor 488-5-dUTP (Invitrogen; C11397; green) and the fragmented genomic DNA of wheat cv. Chinese Spring (300–500bp) as blocking DNA in a ratio of 1:50 per slide.

Slides were counterstained with 4’-6-diamidino-2- phenylindole (DAPI) and analyzed using a high throughput, fully automated Zeiss Axio Imager.Z2 upright epifluorescence microscope (Carl Zeiss Ltd, Oberkochen, Germany). Photographs were taken using a MetaSystems Coolcube 1 m CCD camera. Further slide analysis was carried out using an automated metaphase image capture software, Metafer4, and the ISIS software for image processing (Metasystems GmbH, Altlussheim, Germany).

### Fluorescence *in Situ* Hybridization (FISH)

For multi-color fluorescence *in situ* hybridization (mc-FISH), two repetitive DNA sequences, pSc119.2 (McIntyre et al., 1990) and pAs1 (Rayburn and Gill, 1986), were labeled by nick translation with Alexa Fluor 488-5-dUTP (green) and Alexa Fluor 594-5-dUTP (red), respectively, and hybridized to the slides. Subsequent counterstaining and image capture were performed as described for GISH.

### Genotyping With KASP™ Markers

Genomic DNA was isolated from leaf tissue of 10-day old seedlings in a 96-well plate as described by Thomson and Henry (1995). All lines showing T- and/or D-genome introgressions were genotyped alongside the two durum wheat genotypes, one Ae. tauschii accession P95-81.1.1-1 obtained from USDA and two bread wheat genotypes, Chinese Spring and Paragon, used as controls. The full set of Langdon disomic D-genome substitution lines (obtained from the USDA), were also used as control lines to verify the specificity of the Kompetitive Allele-Specific PCR (KASP) markers to the D-genome. In these lines a pair of D-genome chromosomes substitute a pair of either the A- or the B-genome chromosomes of the same linkage group (Joppa and Williams, 1988).

A total of 80 D-genome specific KASP™ markers (Grewal et al., 2019) of which 29 markers were polymorphic between wheat and *Am. muticum*, were used for simultaneous detection of the D- and T-genome introgression. For each KASP™ marker, two allele-specific forward primers and one common reverse primer were used (**Supplementary Material**). Genotyping reactions were performed in a ProFlex PCR system (Applied Biosystems by Life Technology) in a final volume of 5 µl with 1 ng genomic DNA, 2.5 µl KASP reaction mix (ROX), 0.068 µl primer mix and 2.43 µl nuclease free water. PCR conditions were set as 15 min at 94°C; 10 touchdown cycles of 10 s at 94°C, 1 min at 65–57°C (dropping 0.8°C per cycle); and 35 cycles of 10 s at 94°C, 1 min at 57°C.

Fluorescence detection of the reactions was performed using a QuantStudio 5 (Applied Biosystems) and the data analyzed using the QuantStudio™ Design and Analysis Software V1.5.0 (Applied Biosystems).

## Results

### The Development of Durum Wheat D- and/or T-Genome Introgression Lines

Only 23 seed, out of the 32 randomly selected from the eight WMI parental lines, germinated and reached maturity. Cytogenetic screening *via* mc-GISH showed that 15 of these lines had retained at least one copy of the D-genome introgression. Sixty-three crosses were made between these lines and the two durum genotypes to produce the F_1_ plants. A further 68 back-crosses were made between the F_1_ plants and durum wheat. The total number of crosses, percentage of crosses setting seed, number of seed produced and percentage germination are shown in **Table 2**.

**Table 2:** Number of crosses, percentage of crosses setting seed, number of seeds produced and percentage of seed germination at every generation in the two cross-combination of the WMI lines and their subsequent backcross generations to both the Om Rabi 5 and Karim durum wheat genotypes.

Cross-combination	Generation	Number of crosses	Percentage of crosses set seed	Number of crossed seeds produced	Percentage of germination
WMI line/Om Rabi 5	WMI x Om Rabi 5	28	100%	246	64%
	BC_1_	31	67%	149	87%
	BC_1_-F_2_	**	**	**	93.45%
	BC_1_-F_3_	**	**	**	100%
WMI line/Karim	WMI x Karim	35	82%	242	76%
	BC_1_	37	54%	105	85%
	BC_1_-F_2_	**	**	**	94%
	BC_1_-F_3_	**	**	**	76.90%

Chromosome counts showed that 70% of the BC_1_-F_2_ generation and 88.4% of the BC_1_-F_3_ lines carrying D-genome introgressions had 28 chromosomes. Hence, backcrossing to the durum wheat parent had gradually decreased the average chromosome number through the loss of D-genome univalents.

### Mc-GISH Analysis of the D-Genome Introgression Lines

Only lines carrying D-genome introgressions were selected using mc-GISH at every generation. The percentage of F_1_, BC_1_-F_1_, BC_1_-F_2,_ and BC_1_-F_3_ carrying D-genome introgressions was 33.3, 35.5, 36.5, and 85, respectively, with the percentage retention slightly higher in the lines produced with Karim in all generations except the BC_1_-F_3_ (**Table 3**). The number of D-genome introgressions per line varied between one to three introgressions, with most lines carrying a single D-genome introgression. A higher number of lines carrying inter-genome introgressions were identified in the lines belonging to the G-2 plants, i.e. those in which the initial parental line had one to three introgressions of *Am. muticum*. New D-genome introgressions that were not present in the WMI parental lines were identified in all generations, with the highest number occurring in the BC_1_-F_1_ and BC_1_-F_2_ generations in Karim and Om Rabi 5 cross-combination, respectively (**Table 3**). However, these new introgressions occurred only in the G-2 group and mainly in the progeny from the crosses to Karim.

**Table 3:** Summary result table on the percentage of the retention and occurrence of new D-genome introgressions at the F_1_ and subsequent backcross generations of the WMI lines to Om Rabi 5 and Karim.

Cross-combination	Generation	Number of lines screened	Percentage of lines with a D-genome translocation	Average total chromosome number	Percentage of lines that retained D-genome translocation	Percentage of lines with new D-genome translocation
WMI line/Om Rabi 5	F_1_	50	32%	34	62%	38%
	BC_1_-F_1_	28	35%	31	100%	0%
	BC_1_-F_2_	43	30%	31	46%	54%
	BC_1_-F_3_	9	100%	28	100%	0%
WMI line/Karim	F_1_	54	35%	34	79%	21%
	BC_1_-F_1_	36	36%	30	46%	54%
	BC_1_-F_2_	47	43%	28	85%	15%
	BC_1_-F_3_	41	70%	28	96%	4%

The most frequent introgressions identified initially in the WMI parental lines were D-introgressions into the A-genome, with recombination in the telomeric region (A-d or D-a introgressions—**Table 4**). The newly formed introgressions mainly involved either the D-genome with the A-genome or with both the A- and the B-genomes. Overall, a higher number of different AD (e.g., A-d, d-A-d, D-a, and A.D) and ABD (e.g., D.a-b, D-a-b, D.a-d, and B.a-d) recombinants were identified compared to BD (e.g., B.D and B-d) or AB (e.g., A.B and B-a) recombinants (**Table 4**). A-d recombinants, consisting of a small D-genome segment introgressed into either the long arm (LA) or the short arm (SA) of an A-chromosome, were retained the most between consecutive selfed generations.

**Table 4:** Summary table of the introgressions identified and retained at the F_1_ and subsequent backcross generations of the WMI lines crossed to Om Rabi 5 and Karim genotypes and occurrence of new D-genome introgressions in the G-2 group.

Cross-combination	Generation	Type of D-genomic introgressions retained from previous generation (G-1 and G-2)	Type of the newly formed recombinant chromosomes (G-2)
WMI line/Om Rabi 5	F_1_	A-d_(SA)_, D-a, D-a-b,	D-a-b, A.D, d-A-d, D-a, A.B
	BC_1_-F_1_	A-d_(SA)_, D-a, D-a-b	0
	BC_1_-F_2_	A-d_(SA)_	D-a, D-a-b, A.D, d-A-d, B.D, B-A-d, B-d, B-a-d
	BC_1_-F_3_	A-d_(SA)_	0
WMI line/Karim	F_1_	A-d_(SA)_, D-a, D.a-b, A.D	D-a-b, A.D, B.D, d-A-d
	BC_1_-F_1_	A-d_(SA)_, D-a-b, A.D, d-A-d	A-d_(LA)_, B-d_(SA)_, B-d_(LA)_, D-a, A.B
	BC_1_-F_2_	A-d_(SA)_, A-d_(LA)_, D.a-b, A.D, d-A-d	D-a, B-a
	BC_1_-F_3_	A-d_(SA)_, A-d_(LA)_, D.a-b, A.D, D-a	B-A-d

NB: SA and LA stands for the introgression of the small segment (lowercase letter) in the short or long arm of the chromosome (uppercase letter), respectively. G-1, WMI parental lines without T-genome segment; G-2, WMI parental lines carrying T-genome segments.

The D-genome introgressions identified in the BC_1_-F_2_ progeny from Om Rabi 5 originated from only two BC_1_-F_1_ plants—a G-1 plant and a G-2 plant. Five progenies from the G-1 plant were found to contain a homozygous D-genome introgression of the A-d_(SA)_ type. This introgression was initially identified in the parental hexaploid WMI line and hence, was successfully transferred into Om Rabi 5. Five of the seven progeny from the G-2 BC_1_-F_1_line showed the presence of either a single copy (3 lines) or two copies (2 lines) of a large T-genome introgression. One to three new D-genome introgressions were also identified in all seven lines screened.

The BC_1_-F_2_ Karim lines containing D-genome introgressions were progeny of the same G-2 WMI parental line (BC_3_-F_2_-134). The D-genome introgressions were all telomeric, recombined with an A-chromosome (A-d_(SA)_ or A-d_(LA)_). Ninety-three percent of the lines carrying D-genome introgressions also contained at least one T-genome introgression or chromosome. Hence, simultaneous introgression of the D- and the T-genomes was identified in the tetraploid lines. Mc-GISH showed that the T-genome introgression was recombined with a B-genome chromosome near the telomere (T-b) and had substituted a B-genome chromosome. Homozygous tetraploid introgression lines for both the D- and the T-genomes were identified in the BC_1_-F_3_ generation in some of the selfed progeny of the tetraploid BC_1_-F_2_ lines.

### Genotyping of the Introgression Lines

**Figure 2:**
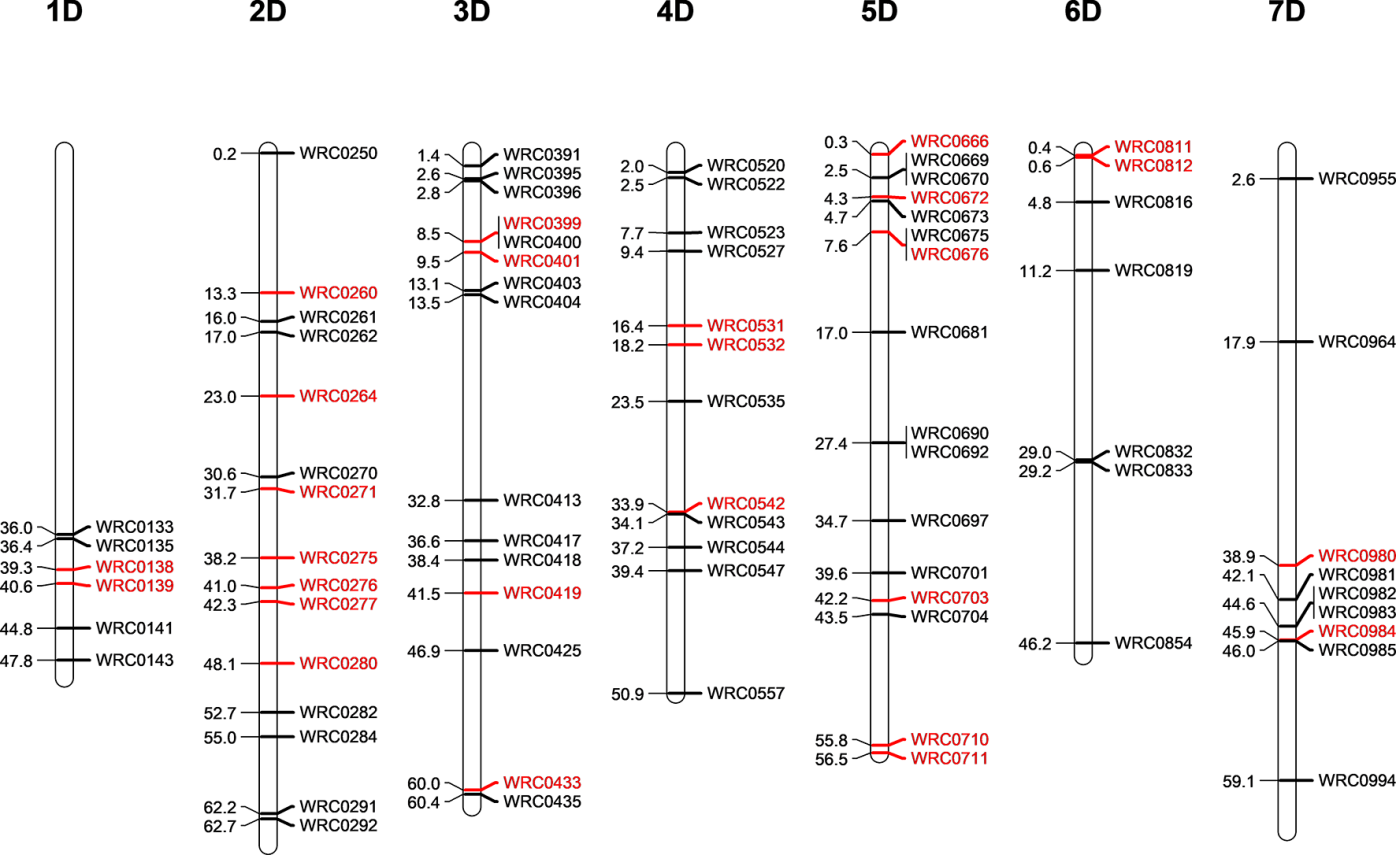
Physical position of the D-genome specific KASP markers on the seven linkage groups of the D-genome of wheat in bp×10^−7^. The D-specific KASP markers that are polymorphic between both wheat and the T-genome of *Am. muticum* (same linkage group as wheat) are highlighted in red.

Five D-genome introgression lines (one in the Om Rabi 5 background and four in the Karim background) were isolated in the BC_1_-F_3_ generation. While the D-genome introgression in Om Rabi 5 background was present in the parental WMI line, the four D-genome introgressions into Karim were identified in four BC_1_-F_1_ lines which were derived from the same WMI line. A total of 80 KASP markers distributed across the seven linkage groups of the D- and T-genomes (except for the 1DS and 1TS arms) were used to characterise both the D-genome and T-genome introgressions in the progenies of the G2-WMI lines (**Figure 2**). A total of 16 D-genome introgression lines were genotyped (including at least two sister lines carrying each of the five introgressions described above).

Genotyping identified the D-genome introgression into Om Rabi 5 as the telomeric region of the 5DS chromosome arm *via* the amplification of the closely linked KASP markers WRC0669 and WRC0670 located at 24,574,003 and 24,971,617 bp (base pair) on wheat chromosome 5D (International Wheat Genome Sequercincy Consortium et al., 2018). However, the absence of amplification of marker WRC0666 (located at 3,031,923 bp) on chromosome 5D indicates that a deletion might have occurred in this region of the 5DS introgressed segment. The introgression was confirmed as homozygous by mc-GISH.

Three of the four D-introgressions into Karim were A-d_(LA)_ introgressions. Two of these segments were characterized as the telomeric region of 1DL and one as the telomeric region of 6DL. KASP markers detected a small difference in segment size between the two 1DL introgressions. Only one marker, WRC0143, located at the telomeric region of 1DL amplified in the two BC_1_-F_3_-202-A and -B sister lines. However, the introgression in the BC_1_-F_3_-214, 215, 312, and 315 sister lines were shown to be larger due to the amplification of WRC0143 and WRC0141. The 6DL introgression was identified as a telomeric segment through amplification of marker WRC0854. The fourth introgression into Karim (A-d_(SA)_) was characterized as the very telomeric region of 2DS *via* the amplification of marker WRC0250.

The polymorphic wheat/*Am. muticum* KASP markers (highlighted in red in **Figure 2**) were able to detect the presence of T-genome introgressions in all the introgression lines from Karim. The two 6DL introgression lines had retained both the 2T and 4T *Am. muticum* introgressions, originally present in the WMI parental line. The remaining lines, however, had retained only the 4T introgression (mc-GISH showed that the large 4T introgression in all these lines had recombined with a B-genome chromosome). Combined analysis with genotyping and mc-GISH identified lines containing simultaneous homozygous introgressions of 4T and either 1DL or 6DL. The introgression remained heterozygous in the BC_1_-F_3_ line analyzed, although the 4T introgression was again homozygous. GISH analysis of the two tetraploid BC_1_-F_3_-324 sister lines showed that the 2T and 4T *Am. muticum* introgressions, identified *via* KASP, were both homozygous substituting two A- and two B-chromosomes. However, these two lines were both sterile and failed to produce seed (BC_1_-F_4_ seed was produced from the rest of the introgression lines).

### Mc-FISH Characterization of the Introgression Lines

**Figure 3:**
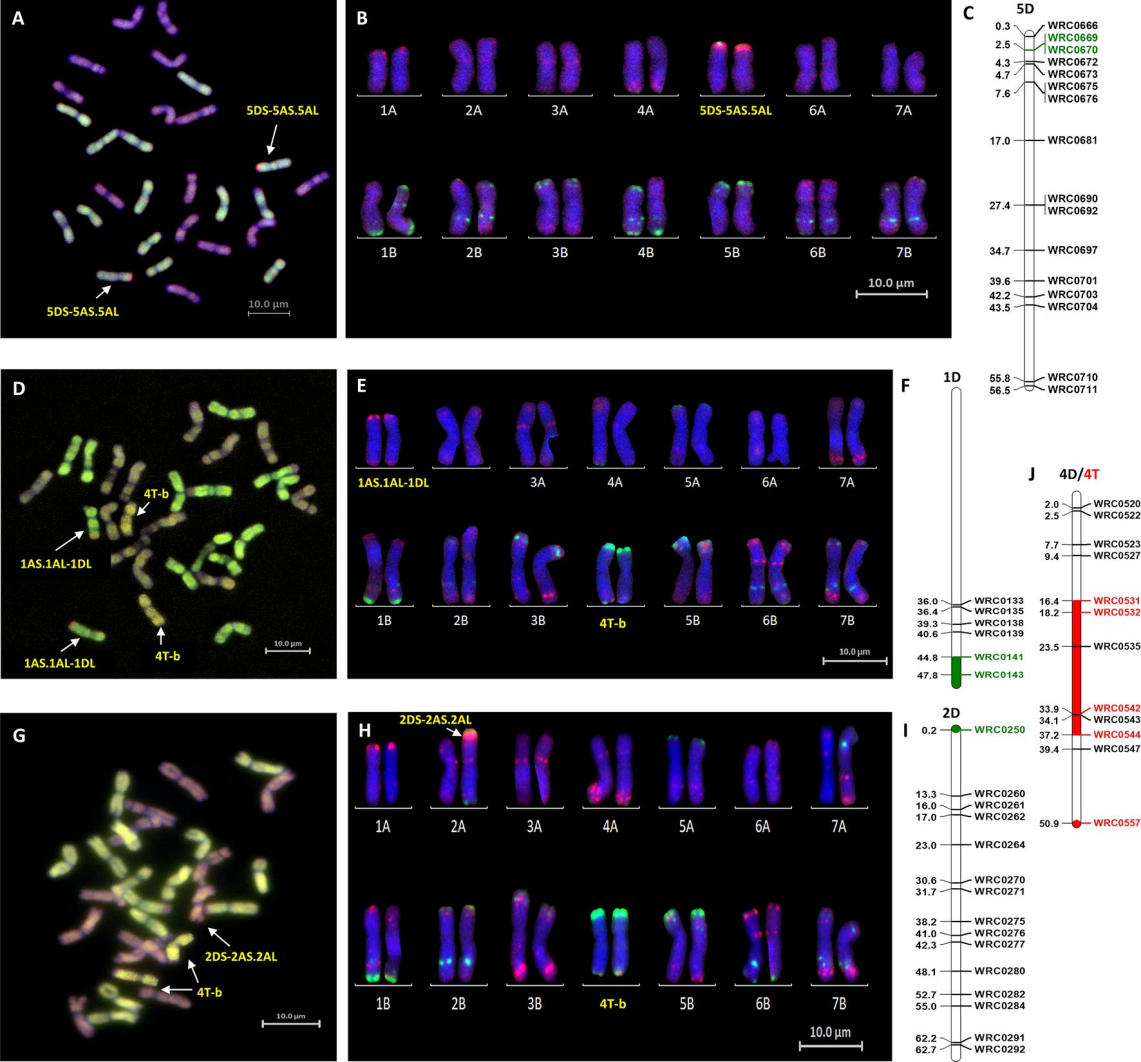
Molecular and cytogenetic characterisation of D-genome and T-genome introgression lines. **(A**, **D**, **G)** Mc-GISH showing the D-genome and T-genome introgression (A-genome in green, B-genome in purple, D-genome in red and T-genome in yellow), **(B**, **E**, **H)** mc-FISH based karyotype using the Oligo-pAs.1 (red) and Oligo-pSc119.2 (green) probes counterstained with DAPI (blue) **(C**,**F**, **I)** physical position (in bp×10^−7^) of the 5DS, 1DL and 2DS introgressions (green markers and region) using D-genome specific KASP markers showing the D-genome introgressions as **(A**, **B**, **C)** 5DS-5AS.5AL in the genomic backgroung of Om Rabi 5, and as **(D**, **E**, **F)** 1AS.1AL-1DL and **(G**, **H**, **I)** 2DS-2AS.2AL in the BC_1_-F_3_-315-E and BC_1_-F_3_-141-A lines, respectively, in the genomic background of Karim. **(J)** characterization of the T-genome introgression as a 4T chromosome recombined in its telomeric long arm with a small B-genome segment noted as 4T-b substituting the pair 4B chromosomes using wheat/T-genome polymorphic KASP markers (red marker and region) in both the BC_1_-F_3_-214-B and BC_1_-F_3_-141-A lines, respectively.

Mc-FISH based karyotyping of the introgression lines was used to identify the wheat chromosomes involved in the introgressions by comparison with the mc-FISH karyotype of Chinese Spring (Tang et al., 2014). Mc-FISH of the homozygous Om Rabi 5 5DS introgression identified it as being recombined with the short arm of chromosome 5A (**Figures 3 A–C**). Only two of the D-genome introgressions into Karim could be characterized as the 1DL introgression identified in the BC_3_-F_2_-202 sister lines and the 6DL introgression were too small to detect. The 1DL introgression identified in the BC_1_-F_3_-214, 215, 312, and 315 sister lines, however, was recombined with the long arm of chromosome 1A (**Figures 3 D–F**) and the 2DS introgression with the short arm of chromosome 2A (**Figures 3 G–I**). The B-genome introgression, recombined with the large 4T introgression, was also too small to detect. However, this single or homozygous 4T-b recombinant chromosome was found to have substituted either a single or a pair of 4B chromosomes (**Figures 3 E–J**).

## Discussion

Pentaploid crosses between bread and durum wheat have previously been shown to generate viable F_1_ seed that can be used in a backcrossing programme to either of the parents (Eberhard et al., 2010; Martin et al., 2013; Kalous et al., 2015). The presence of inter-genomic rearrangements in the hexaploid background of wheat/wild relative introgression lines [such as those identified in wheat/*Am. muticum* introgression lines by King et al. (2017)] can thus be used for the introgression of the D-genome of bread wheat into durum wheat. While the overall aim of the current programme was to introgress the D-genome, the crosses also had the potential to increase the genetic variability of the durum A- and B-genomes through recombination with their homologues of bread wheat.

In crosses involving parents of different ploidy levels, it has been shown that using the higher ploidy level genotype as the maternal parent is generally more successful in producing viable F_1_ progeny (Ramsey and Schemske, 1998; Kalous et al., 2015). In pentaploid wheat crosses, the hexaploid parent is usually used as the female parent (Padmanaban et al., 2017a; Padmanaban et al., 2017b) and thus, the hexaploid WMI lines were used here as the female parent. Viable F_1_ seeds were obtained with both durum wheat parents, Om Rabi 5 and Karim. However, a higher seed set was obtained in the F_1_ and the BC_1_ generations using Om Rabi 5 as compared to Karim suggesting a higher crossing compatibility of the Om Rabi 5 genotype in the crosses with the bread wheat used here. Other studies have highlighted the importance of the parental choice, as well as the direction of the cross, in the production of viable pentaploid hybrids (reviewed in Padmanaban et al., 2017b).

Mc-GISH was used to visualize the three genomes of wheat and thus, the presence of inter-genomic recombinant chromosomes within the wheat genome of the original introgression lines. This technique has been widely used for studying genome rearrangements, alien introgressions and the discrimination between different genomes in polyploid cereals (Schwarzacher et al., 1989; Schwarzacher et al., 1992; Schubert et al., 2001; Silva and Souza, 2013).

A higher number of lines carrying D-genome introgressions were distinguished in the progeny of Karim in all generations as compared to Om Rabi 5. This could indicate a higher tolerance of Karim to the presence of the D-genome introgressions emphasizing the importance of the durum parent selected for the work. However, a higher seed set with Om Rabi 5 does indicate that the choice of durum parent might not be straight forward. In addition to the D-genome introgressions present in the parental WMI lines, new wheat sub-genome introgressions involving the D- with either the A- and/or the B-genome and the A- with the B-genome were identified at all generations from the F_1_ to the BC_1_-F_2_. A mc-FISH karyotype [based on the karyotype for Chinese Spring developed by Tang et al. (2014)] was used to identify the wheat chromosomes present in the tetraploid BC_1_-F_3_ plants containing single or homozygous D-genome introgressions from both cross combinations. This showed the presence of a pair of 5B chromosomes in all lines. Since wild-type wheat (Paragon) was used to develop the WMI parental lines, instead of a *ph1b* mutant wheat (King et al., 2017), the inter-genomic rearrangements that occurred in the later generations were not due to the absence of the *Ph1* gene. However, the presence of more than two genomes (A, B, D, and T) and unequal chromosome numbers in one cell could have promoted abnormal meiotic behavior leading to homologous paring. Wheat chromosomes in the selfed progeny of wheat/rye monosomic addition lines, such as 1R, 4R and 6R, show abnormal behavior at meiosis resulting in the elimination or the addition of some of the wheat chromosomes e.g., three 4A-chromosomes were observed in one of the progeny from a 7R monosomic addition line and chromosomes 5A and 4B were eliminated from some of the progeny of the 6R monosomic addition line in addition to alterations of the wheat chromosomes (Fu et al., 2012; Fu et al., 2013).

In the present study, new recombinant events occurred only in lines belonging to the G-2 group with at least one large T-genome introgression/chromosome present in the parental lines. *Am. muticum* is known to contain genes that promote pairing between homologous chromosomes/suppress the effect of the *Ph1* gene in hybrids with allopolyploid wheat (Dvorak, 1972; Dover and Riley, 1972). Similarly, two major *Ph1* suppressor loci, *Su1-Ph1* and *Su2-Ph1* were mapped on the distal end of the long arm of chromosomes 3S and 7S, respectively, in *Ae. speltoides* (Dvorak et al., 2006; Li et al., 2017). It may be possible that some of the introgressed segments from *Am. muticum* also carry a *Ph1* suppressor gene. However, new introgressions were distinguished in the progeny of G-2 WMI lines carrying different introgressions of *Am. muticum* such as 2T, 4T, 6TS.7TL and 1TS.3TL. Hence, it is possible that the stress caused by the presence of *Am. muticum* introgression(s) might be one factor inducing recombination.

The new inter-genomic rearrangements were found to be made up of 80% D-genome with either the A- and/or the B-genome. The univalent state of the D-chromosomes in these lines may also have promoted the rearrangements. The A- and B-genomes have previously been shown to be more similar to the D-genome than they are to each other (Marcussen et al., 2014). Pairing is frequently observed between the A- and the D-genomes in wheat-rye hybrids denoting a much lower differentiation between these two genomes than between the A- and B- or B- and D-genomes, at least in the regions of high recombination in the distal chromosome regions (Naranjo et al., 1987; Marcussen et al., 2014). This is consistent with the high level of A-D recombinant chromosomes observed in the present study, especially in the telomeric regions of the chromosomes. For example, for the introgressions that could be identified with mc-FISH, analysis showed that the slightly larger 1DL introgression had recombined with the short arm of 1A and the 2DS introgression with the short arm of 2A.

Only the small telomeric D-genome introgressions were successfully transferred into the tetraploid background of both durum wheat varieties indicating that introgressions of a smaller size have a higher chance of being transmitted compared to larger D-genome introgressions. If the large D-genome segments do not have the ability for genetic compensation for the homologous A- or B- genome chromosome segments, it less likely they will be retained. The inter-genomic recombinant chromosomes that were present as additions were generally lost due to a lack of pairing at meiosis. For instance, the A-d translocation when present as a monosomic addition, in the tetraploid background with 29 chromosomes, was not retained after self-fertilisation. Whereas, the recombinant chromosomes that had substituted one of the wheat chromosomes had a higher rate of retention and transmission.

KASP marker analysis showed that the *Am. muticum* introgression in all the Karim D-genome introgression lines was a large 4T introgression previously confirmed as present in the WMI parental line, together with a 2T introgression, using the Axiom^®^ Wheat-Relative Genotyping Array (King et al., 2017). The 4T introgression was highly retained in the progeny of Karim. Lines homozygous for both 4T and 1DL were identified in the BC_1_-F_3_ where FISH analysis showed that the pair of 4T recombinant chromosomes were substituting the pair of 4B-chromosomes. Under glasshouse conditions, these introgression lines were fertile with a normal spring wheat growth cycle and a durum wheat head type. Thus, the disomic 4T-b(4B) substitution did not affect fertility in these lines. Among the full set of Chinese Spring nullisomic–tetrasomic lines, only the 4B nullisomic tetrasomic line (N4BT4D) was completely male sterile (and had to be maintained as a monosomic tetrasomic line, M4BT4D) suggesting the presence an essential gene for male fertility on this chromosome (Sears, 1966). In addition, Endo and Gill (1996), failed to establish a homozygous deletion line for the short arm of chromosome 4B in a hexaploidy background, because plants were male sterile. However, Langdon durum 4D(4B) disomic substitution line is also fertile and can be selfed in the absence of the 4B chromosomes (Joppa and Williams, 1988). Hence, the 4D disomic substitution compensates for the absence of both copies of chromosome 4B at the tetraploid level but does not compensate when present as tetrasomic in the 4B nullisomic tetrasomic line at the hexaploid level. This can possibly be due to the interaction of several genes. Similar to the Langdon durum 4D(4B) disomic substitution line, the 4T-b introgression fully compensate for the absence of the male fertility gene, *Ms1* (Driscoll, 1975), on chromosome 4B in durum wheat.

To our knowledge, this is the first study to transfer D-genome introgressions into either the A- or B-genomes, present in hexaploid wheat/wild relative introgression lines, into durum wheat. Advances in cytology and mc-GISH have made it possible to identify, characterize and track these genome rearrangements, together with wild relative introgressions, enabling their transfer *via* pentaploid crosses. Mc-GISH, however, is labour intensive and relatively low throughput. KASP markers, able to detect the presence of *Am. muticum* introgressions in wheat, have been developed at the WRC. Many of the KASP markers are wheat genome specific and those that are specific to the D-genome were used for the detection of the D-genome introgressions in the later generations. However, for future work, these markers will be used in the earlier generations such as the F_1_ and BC_1_-F_1_ with the mc-GISH analysis used for validation and chromosome counting in the later generations. The developed introgression lines can be of use in durum wheat breeding through marker assisted selection, to screen for several traits of interest such as disease resistance or agronomic traits. Once multiplied, D- and T-genome introgression lines as well as the KASP markers associated with the introgressed segments will be made freely available upon request from the GRU.

## Data Availability

The raw genotyping data supporting the conclusions of this manuscript will be made available by the authors, without undue reservation, to any qualified researcher.

## Author Contributions

MO conducted the crossing program. MO and CY performed the *in situ* hybridization experiments. MO, SH-E, DS, and SA performed the genotyping analysis. MO, JK, IK, and SG wrote the manuscript. MO, AY, PG, PS, IK, and JK conceived the experimental design. All authors read and approved the final manuscript.

## Funding

This study was supported by the Monsanto Beachell Borlaug International Scholarship program (2015), The Crop Trust and the Biotechnology and Biological Sciences Research Council [grant number BB/P016855/1] as part of the Designing Future Wheat programme (DFW). The funding body played no role in the design of the study and collection, analysis and interpretation of data and in writing the manuscript.

## Conflict of Interest Statement

The authors declare that the research was conducted in the absence of any commercial or financial relationships that could be construed as a potential conflict of interest.

## Abbreviations

WMI, wheat/Am. muticum introgression; SA, short arm; LA, long arm.

## Supplementary Material

Table S1 Primer sequences for all KASP markers used in this study.
